# Gram-negative central line-associated bloodstream infection incidence peak during the summer: a national seasonality cohort study

**DOI:** 10.1038/s41598-022-08973-9

**Published:** 2022-03-25

**Authors:** Koen Blot, Naïma Hammami, Stijn Blot, Dirk Vogelaers, Marie-Laurence Lambert

**Affiliations:** 1 Department of Epidemiology and Public Health, Sciensano, Brussels, Belgium; 2 Faculty of Medicine and Health Sciences, Ghent University, Ghent, Belgium; 3 Healthcare-Associated Infections and Antimicrobial Resistance, Public Health and Surveillance Department, Sciensano, Brussels, Belgium; 4 Agentschap Zorg en Gezondheid, Vlaamse Overheid, Ghent, Belgium; 5 Service des Soins de Santé, Institut National d’Assurance Maladie-Invalidité, Brussels, Belgium; 6 General Internal Medicine, Ghent University Hospital, Ghent, Belgium; 7 Burns, Trauma and Critical Care Research Centre, Centre for Clinical Research, Faculty of Medicine, The University of Queensland, Brisbane, Australia

**Keywords:** Bacterial infection, Epidemiology, Disease prevention

## Abstract

Central line-associated bloodstream infections (CLABSI) cause increased morbidity, mortality, and hospital costs that are partially preventable. The phenomenon of seasonality among CLABSI rates has not been fully elucidated, but has implications for accurate surveillance and infection prevention trials. Longitudinal dynamic cohort of hospitals participating in hospital-wide and intensive care unit bloodstream infection surveillance for at least one full year over 2000 to 2014. Mixed-effects negative binomial regression analysis calculated the peak-to-low ratio between months as an adjusted CLABSI incidence rate ratio (IRR) with 95% confidence intervals (CI). Multivariate regression models examined the associations between CLABSI pathogens and ambient temperature and relative humidity. The study population included 104 hospital sites comprising 11,239 CLABSI. Regression analysis identified a hospital-wide increase in total CLABSI during July–August, with a higher gram-negative peak-to-low incidence rate ratio (IRR 2.52 [95% CI 1.92–3.30], p < 0.001) compared to gram-positive bacteria (IRR 1.29 [95% CI 1.11–1.48], p < 0.001). Subgroup analysis replicated this trend for CLABSI diagnosed in the intensive care unit. Only gram-negative CLABSI rates were associated with increased temperature (IRR + 30.3% per 5 °C increase [95% CI 17.3–43.6], p < 0.001) and humidity (IRR + 22.9% per 10% increase [95% CI 7.7–38.3), p < 0.001). The incidence and proportion of gram-negative CLABSI approximately doubled during the summer periods. Ambient temperature and humidity were associated with increases of hospital-acquired gram-negative infections. CLABSI surveillance, preventive intervention trials and epidemiological studies should consider seasonal variation and climatological factors when preparing study designs or interpreting their results.

## Introduction

Central venous catheters (CVC) are necessary for medication, fluid, and blood product infusion, and hemodialysis. Unfortunately, these invasive devices can lead to preventable central line-associated bloodstream infections (CLABSI)^1^. CLABSIs can lead to septic shock, hematogenous bacterial seeding with organ infection, increased length of hospitalization and costs, which impact morbidity and prognosis^2^. CLABSI rates are primarily dependent on exposure risk: central line catheterization and duration. Considering this exposure, other risk factors include catheter type, insertion site and infection prevention practices. Reported infection rates for non-tunneled short-term CVCs range around 4.4 per 100 catheters, 2.7 per 1000 catheter-days and 1.6 per 10,000 patient-days, but depends on quality of care with adherence to preventive hygienic measures^3,4^.

Infection seasonality has been well documented for influenza and more recently among bacterial infections. Increased summer incidence has been documented for gram-negative infections^5,6^, BSI^7–15^, hospital-acquired infections^5,6^, peritoneal dialysis-related infections^16,17^, intra-abdominal^18^ and surgical site infections^19,20^. One study has identified seasonality for in-hospital CLABSI rates^21^. CLABSI are largely preventable and can be deduced to human errors with breaks in appropriate aseptic measures during catheter manipulation^22,23^. This complicates the interpretation of CLABSI seasonality compared to influenza, which has been linked to crowding and climate variables.

The reason for bacterial seasonal fluctuations has not been fully elucidated. Seasonal variation may be a phenomenon manifested by changes and interactions between patient, pathogen, environmental and hospital factors. Proposed explanations include seasonal changes in human behavior such as recreational water exposure, water consumption, or food preparation^24^, temperature^6,15,25^, humidity^15^, moisture within air-conditioned temperature-controlled environments^5^, and decreased hospital personnel (e.g. nurse-to-patient ratio) which predisposes to non-adherence to infection prevention guidelines^25–27^.

The Belgian *Surveillance of Bloodstream Infections in Hospitals* program has collected hospital-wide HABSI case-based data since 1992 to improve nationwide surveillance of these severe bacterial infections^4^. The study objective was to examine the presence of seasonal variation of hospital-wide CLABSI incidence including regression analysis for climatological variables and subgroup analysis per pathogen and CLABSI diagnosed in the intensive care unit (ICU).

## Methods

This manuscript is reported according to the STROBE guidelines. All methods were carried out in accordance with relevant guidelines and regulations. The Belgian royal decree of April 2002 mandated bloodstream infection surveillance in acute care hospitals. The committee of Social Security and Health (SCSZG/16/245, deliberation nr. 16/111 of December 20, 2016) approved this surveillance. Informed consent was waived by this same committee.

### Study design and setting

A national cohort study of CLABSI epidemiology was performed based on the Belgian *Surveillance data of BSI in Hospitals* program from January 2000 to December 2014. The full protocol, revised in 2013, is available online in Dutch and French^28^.

### Participants

Participation in the surveillance program entails case-based recording of all CLABSI for a minimum of one trimester per year. Participation was voluntary, but became mandatory in 2014. Eligible hospitals were those with more than 150 beds. To analyze seasonal variation, hospitals included in the cohort performed surveillance for CLABSI (numerator) with hospital-wide patient-day (denominator) data for a full year, i.e. four trimesters within a calendar year, during at least one year.

### Case definitions and variables

A laboratory-confirmed BSI required at least two separate samples if the causal micro-organism is a skin commensal and one sample in case of a recognized pathogen (Appendix 1). HABSI were those not present or incubating at the time of admission to the acute care setting (≥ 48 h). An episode in the same patient caused by the same microorganism was considered to be a novel occurrence if there were 14 days between the two episodes. During 2000–2012, CLABSI diagnosis could be classified as confirmed or probable. A definitive CLABSI diagnosis required a concomitant positive CVC tip with identification of the same BSI microorganism (Appendix 2). Probable CLABSI consisted of HABSI with a CVC in place, not secondary to another infection, and considered by the clinician to originate from the CVC. To avoid overdiagnosis, BSI of unknown origin with a central line in place the previous 48 h were not automatically classified as CLABSI unless there was suspicion of catheter infection. Denominator data included number of hospital-wide and ICU patient-days per trimester. CLABSI incidence was reported as a rate per 10 000 patient-days or per 1000 ICU patient-days.

### Statistical methods

Mixed-effects negative binomial distribution regression model calculated the adjusted incidence rate ratio (IRR) with 95% confidence intervals (CI) for monthly CLABSI rates. Month was analyzed as a categorical variable, which allows for the identification of a peak-to-low ratio between two months. The comparison of the lowest to the peak incidence rate between months describes the amplitude of the seasonal pattern as an IRR^29^. This peak-to-low IRR is equal to the peak month’s incidence rate per patient-days divided by the lowest month’s incidence rate. The analysis was performed hospital-wide and stratified for gram-positive, gram-negative and fungal CLABSI. Fixed effects included year, university-affiliated status, hospital bed size, and infection risk exposure expressed as monthly patient-days. Hospitals were applied as random effects to account for varying hospital participation and heterogeneity. The number of patient-days was reported per trimester, but to calculate monthly patient-day risk exposure and incidence rate analysis, patient day trimester data was averaged by dividing by three. Monthly peak-to-low IRR results of the regression analysis predicted monthly CLABSI rate estimates, which were used to graph monthly seasonality by microorganisms. This way both the relative (IRR), absolute changes (mean incidence rate per patient-days), and microorganism proportions (percentage cultured gram-negative bacteria over total CLABSI) could be reported. Sensitivity analysis included separate assessment of CLABSI from 2000 to 2012 that fulfilled the criteria of a definitive diagnosis with concomitant positive catheter tip culture. Subgroup analysis replicated this for CLABSI diagnosed in the ICU.

To assess the climate influence on the infection rate, monthly average ambient temperature (°C), relative humidity (%) and precipitation (mm) from 2000 to 2014 were collected from a meteorological online system based in the center of Belgium^30^. Two mixed-effects negative binomial regression models were applied to examine the association between CLABSI and climate variables, adjusting for university-status and infection risk exposure. One applied season, temperature, and humidity as fixed effects to assess meteorological influence on CLABSI across all seasons. The second examined weather influences within seasons by applying temperature and humidity-by-season interaction terms^15^. Seasons were defined as winter (December–February), spring (March–May), summer (June–August), and autumn (September–November). These models were applied to hospital-wide total, gram-positive, gram-negative and fungal CLABSI.

All statistical analyses were done using Stata software, version 14. The mixed-effects negative binomial regression analysis was performed with the *menbreg* function. Statistical significance was set at p ≤ 0.05.

### Results

Surveillance from 2000 to 2014 led to the identification of 11,239 CLABSI with 12 401 cultured microorganisms. The cohort of hospitals performing surveillance for an entire year consisted of 104 hospital sites (Table 1). This cohort included 7816 CLABSI in 7563 patients with a total of 8618 cultured microorganisms. Selection of hospitals performing surveillance consecutively for an entire calendar year led to exclusion of 3420 (30.4%) CLABSI. Missing patient-day data only led to a further loss of 3 CLABSI (0.1%) hospital-wide. For the subgroup analysis, missing ICU patient-day data (314 of 3299) was interpolated based on the median number of monthly ICU patient-days. The median CLABSI rate was 1.0 (IQR 0.5–1.6) per 10 000 patient-days, with an ICU CLABSI rate of 1.04 (IQR 0.51–1.62) per 1000 patient-days.

**Table 1:** Central line-associated bloodstream infections and microorganism incidence per trimester among 104 participating hospital sites.

Measurement	Surveillance trimester				
	Jan–Mar	Apr–Jun	Jul–Sept	Oct–Dec	Total
CLABSI	1808	1789	2226	1993	7816
Gram-negative	338	377	584	446	1745
Gram-positive	1422	1359	1594	1493	5868
Fungal	214	206	272	232	924
Patient-days	13,647 297	12,877 482	12,221 416	13,326 742	52,072 936
Patient admissions	1,820,767	1,741,661	1,636,991	1,784,115	6,983,534
Mean CLABSI rate	1.32	1.39	1.82	1.50	1.50
Temperature	4 (3–6)	11 (8–14)	18 (17–19)	11 (9–15)	11 (6–16)
Relative humidity	87 (83–89)	73 (70–76)	74 (71–77)	84 (80–88)	79 (74–90)

Quarterly data on hospital-wide mean CLABSI rates per 10 000 patient-days. The rate of total CLABSI classifies polymicrobial BSI as a single CLABSI. *CLABSI* central line-associated bloodstream infection.

Gram-positive pathogens constituted the majority of cases, followed by gram-negative and fungal microorganisms (Table 2). The most common were coagulase-negative staphylococci, *Staphylococcus aureus*, and *Candida* spp. Less common microorganisms included Enterobacterales, *Acinetobacter* spp., and viridans group streptococci. *Enterococcus faecium* consisted of 19.1% of all enterococcal infections. 12.1% of CLABSI were polymicrobial. During 2000–2012 CLABSI were classified as definitive or probable. This period accounted for 7533 CLABSI, among which 3492 (46.4%) were classified as definitive with positive catheter tip culture.

**Table 2:** Proportions of central line-associated bloodstream infection microorganisms.

Microorganisms	Number	(%)
Gram-positive	5646	(68.1)
CNS	3838	(47.3)
* S. aureus*	1201	(14.8)
* Enterococcus* spp.	432	(5.3)
Gram-negative	1679	(20.2)
* Enterobacter* spp.	365	(4.5)
* Klebsiella* spp.	355	(4.5)
* P. aeruginosa*	237	(2.9)
* E. coli*	219	(2.7)
* Acinetobacter* spp.	156	(1.9)
*Candida* spp.	889	(10.7)

*CNS* coagulase-negative staphylococci.

**Figure 1:**
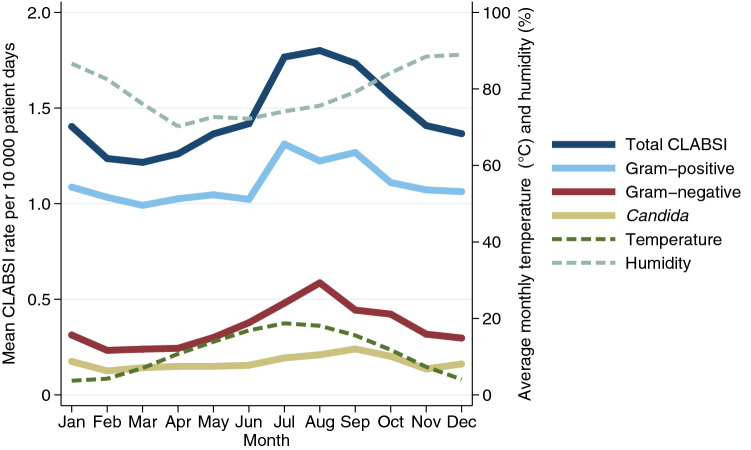
Seasonal variation of hospital-wide central line-associated bloodstream infection incidence, per microorganism. Composite monthly incidence rates of central line-associated bloodstream infection (CLABSI) based on the hospital-wide mixed-effects regression analysis results (Table 3). Total CLABSI seasonality during July–August was associated with both gram-positive and gram-negative increases. The rate of total CLABSI classifies polymicrobial BSI as a single CLABSI.

Mixed-effects regression analysis identified a sinusoidal pattern with an approximately 50% increase in total CLABSI from February up to August (Table 3). Seasonal variation was most pronounced among gram-negative microorganisms; when comparing August to March there was a two-and-a-half-fold increase in incidence rate during August (IRR 2.52 [95% CI 1.92–3.30], p < 0.001, Fig. 1). Although the gram-positive IRR increased significantly, this relatively small incidence rate ratio (IRR 1.29 [95% CI 1.11–1.48], p < 0.001) translated into an absolute rate increase of approximately 0.25 HABSI per 10,000 patient-days during the months of July–September. The change in relative IRR was statistically significant for fungal CLABSI, yet the absolute rate change was negligible. Even with this combined increase of total CLABSI during the summer months, a proportionate increase in the percentage of CLABSI in favor of gram-negative pathogens remained. Gram-negative bacteria represented 18.8% of total CLABSI in February, and this percentage increased to a maximum of 32.6% in August. Subgroup analysis of CLABSI diagnosed in the ICU also recognized gram-negative CLABSI seasonality with increasing incidence over the summer months which peaked during October (Appendix 3).

**Table 3:** Mixed-effects regression analysis of peak-to-low incidence rate ratio seasonality.

CLABSI	IRR	95% CI	p-value	Low month	Peak month
Total	1.47	1.29–1.67	< 0.001	March	August
Gram-positive	1.29	1.11–1.48	< 0.001	March	July
Gram-negative	2.52	1.92–3.30	< 0.001	February	August
*Candida* spp.	1.89	1.34–2.66	< 0.001	February	September

Mixed-effects negative binomial regression model that calculated the adjusted incidence rate ratio (IRR) using hospital units as random effects and other covariates such as year, month and university-affiliation as categorical fixed effects. Incidence rate ratios are expressed as a peak-to-low ratio between the lowest incidence rate to the respective peak month per pathogen.

Across 2000–2014, the spread (5th to 95th percentile) of average temperature across all months was 2.4 °C to 19.3 °C (median 11 °C) and 67% to 90% for relative humidity (median 79%). When assessing the impact of meteorological factors on CLABSI incidence rates over all seasons, a significant association was found between temperature and the total CLABSI rate. Notably, subgroup analysis revealed that this relation was due to the gram-negative pathogens. Gram-negative CLABSI demonstrated IRR increases of 30.3% (17.3–43.6, p < 0.001) per rise of 5 °C ambient temperature across all seasons. Except for during winter, this association was present within all seasons, which represents an incidence rate difference between a warm versus a cool summer (Table 4).

**Table 4:** Associations between hospital-wide central line-associated bloodstream infections and weather, year-long and by season.

CLABSI type per climate	IRR %, (95% CI) for CLABSI, per microorganism				
	All seasons	Winter	Spring	Summer	Autumn
**Temperature**					
Total	12.1 (6.1–18.1)*	− 12.8 (− 26.5–1.34)	10.5 (2.0–19.1)*	17.1 (3.6–30.8)*	17.4 (10.0–24.8)*
Gram-positive	6.3 (− 0.4–13.1)	− 13.3 (− 28.9–2.7)	3.2 (− 6.3–12.9)	14.1 (− 1.3–30.1)	11.8 (3.4–20.3)*
Gram-negative	30.3 (17.3–43.6)*	− 10.5 (− 40.2–21.2)	27.0 (7.7–47.0)*	41.2 (13.8–70.0)*	39.2 (23.4–55.4)*
*Candida* spp.	17.9 (2.0–34.3)*	− 27.8 (− 63.0–10.2)	15.3 (− 7.5–39.2)	2.3 (− 33.0–40.2)	28.6 (8.9–49.1)*
**Humidity**					
Total	4.6 (− 2.5–11.7)	8.9 (1.5–16.3)*	5.4 (− 2.7–13.7)	3.8 (− 4.0–11.7)	4.9 (− 2.1–11.9)
Gram-positive	1.3 (− 6.8–9.5)	5.6 (− 2.8–14.0)	3.9 (− 5.4–13.3)	0.2 (− 8.9–9.3)	2.3 (− 5.6–10.2)
Gram-negative	22.9 (7.7–38.3)*	30.3 (14.1–46.8)*	25.4 (7.5–43.6)*	21.7 (5.6–38.0)*	23.2 (8.0–38.6)*
*Candida* spp.	− 4.8 (− 23.6–14.3)	− 0.5 (− 19.9–19.2)	− 8.9 (− 30.2–12.9)	− 2.2 (− 23.6–19.7)	− 8.5 (− 26.6–9.9)

Estimates of the mixed-effects negative binomial regression model, adjusted for university-status and infection risk exposure. All season model controls for season; seasonal models assessed weather-by-season interaction term. Adjusted incidence rate ratios (IRR) are expressed as a percentage change with 95% confidence intervals per increase of 5 °C average temperature or 10% relative humidity. *CLABSI* central line-associated bloodstream infection. *P-value significant (≤ 0.05).

Likewise, increases in 10% relative humidity was associated with increased gram-negative (IRR 22.9%, 7.7–38.3, p < 0.001), but not gram-positive nor fungal CLABSI rates. As with temperature, humidity’s role in gram-negative incidence rates was further demonstrated within all separate seasons including the winter. Precipitation was not associated with CLABSI incidence. Analysis of CLABSI with a definite diagnosis affirmed the seasonal variation among total, gram-positive and gram-negative CLABSI (Appendix 4).

## Discussion

This cohort study analyzed CLABSI seasonality over 15 years. Hospital-wide total CLABSI exhibited sinusoidal seasonal variation with rising rates from March to August. Gram-positive CLABSI demonstrated slightly higher rates during the summer to autumn period; yet the most marked rise was among gram-negative pathogens, which displayed both an increased absolute rate change and proportion out of total CLABSI. Gram-negative bacteria were responsible for less than one fifth of CLABSI and this percentage increased to nearly one third during the August summer peak. Sensitivity analyses confirmed these findings when examining CLABSI with a definitive diagnosis by catheter tip culture or paired peripheral and CVC blood cultures. Climate variables of ambient temperature and humidity were shown to be associated with the total in-hospital CLABSI incidence rate after adjustment for hospitals and seasons. Gram-negative bacteria were the only family of microorganisms to consistently exhibit associations with temperature and humidity. Subgroup analysis of CLABSI diagnosed in the ICU also demonstrated gram-negative increases during the summer with a delayed peak during October.

Strengths of this study include long-term surveillance over multiple different hospitals, detailed microorganism identification, sensitivity and subgroup analyses, and mixed-effects regression analysis for adjusted peak-to-low monthly IRRs and climatological variables^15,29^.

Main limitations included lack of patient comorbidity, number of catheter-days, data on nurse-to-patient ratio and in-hospital climate data. Furthermore, the assumption was made that reported quarterly data on patient-days was equally distributed across the calendar months. Another known limitation is the diagnostic difficulty of CLABSI, with bloodstream infections falsely attributed to the catheter instead of occult gram-negative intra-abdominal abscesses or mucosal barrier injury. Nonetheless, our findings were reproduced among subgroup analysis for CLABSI diagnosed with catheter tip or paired blood cultures.

Because of insufficient data on number of catheter-days, number of patient-days had to be used to adjust for different risk exposure. Fluctuations in device-usage or nurse-staffing between seasons may influence the CLABSI incidence rate. Nonetheless, higher ambient temperature within the summer season remained significantly positively associated with CLABSI rates. Furthermore, although relative humidity was lower during the summer it was still positively associated with higher gram-negative CLABSI rates.

Although much of the literature has focused on CLABSI prevention, little has been reported on seasonality of these hospital infections^27,31^. Multiple previous studies have identified seasonal variation with summer increases in gram-negative BSI, but not fully examined HABSI nor BSI per infectious focus^14,15^. Peritoneal dialysis catheter-related peritonitis have demonstrated increased gram-negative IRRs during warmer months and gram-positive bacteria during the spring^16,17^. Other studies have shown summer increases in gram-negative outpatient healthcare-associated BSI, among which a large proportion were associated with intravascular catheters^13,14,32,33^. Higher risk for gram-negative BSI development has been linked to climate, such as geographic proximity to the equator, even after adjusting for healthcare expenditure differences^26^. Higher gram-negative BSI have been found during warmer months^5,6,11^ along with temperature associations^6,15,26^.

However, these studies did not focus on CVCs, hospital-acquired or inpatient catheter infections, or CLABSI due to different microorganisms. One recent study within a European tertiary-care hospital demonstrated a CLABSI incidence increase during the summer period over two surveillance years^21^. Unlike this study, theirs described an association with precipitation and not temperature nor humidity. However, limitations included a short-term duration and that no stratification per microorganism species could be performed.

This is the first large study to describe an increased gram-negative inpatient CLABSI incidence during the summer and association with climatological factors. Previous studies have found higher gram-negative BSI during warmer months^5,6,11^ with temperature associations^6,15,26^, but did not perform CLABSI subgroup analysis.

How increases in ambient temperature and humidity may translate to higher CLABSI rates is not fully elucidated. Disruption of the sterile catheter dressing has previously been defined as an important risk factor in the pathogenesis of CLABSI through colonization of the external lumen^34^. During the summer the warmer, damper skin environment may predispose to catheter dressing disruption. This could then lead to bacterial catheter colonization and subsequently CLABSI^35^. Combined with a decreased nurse-to-patient ratio, higher device-utilization rates, and frequent or inadequate catheter dressing changes, the summer climate changes may lead to an increased CLABSI rate^27,31,35^. However, even though the majority of CLABSI are skin commensals, the regression analysis did not identify an association between climate and gram-positive CLABSI.

Multidrug-resistant gram-negative bacteria have been described as waterborne pathogens causing healthcare-associated infections, commonly linked to contaminated sinks as a reservoir^36^. A narrative review found that 9.7% to 68.1% of random ICU water samples were positive for *P. aeruginosa*, and between 14.2 and 50% of patient infections were due to genotypes found in ICU water^37,38^. The summer months may improve the growth and density of gram-negative flora, leading to higher colonisation pressure among humans or the environment, increasing the risk of catheter colonization and CLABSI development.

This is the first multicenter cohort to identify seasonal variation of gram-negative CLABSI. These findings have important implications on multiple facets concerning infection prevention and surveillance. New trials investigating CLABSI prevention interventions should account for seasonal variation as a confounding factor, since implementation during a peak summer month could lead to an infection rate decrease falsely attributed to the intervention instead of regression to the mean^1^. CLABSI surveillance monitoring solely during low incidence rate months should be avoided; surveillance sampling during random trimesters has been validated as an accurate method for incidence measurement^39^. Although quality improvement strategies should be continuously performed, new initiatives could be started during peak incidence months where there may be more room for improvement^40^. CLABSI documentation has become an important quality of care indicator due to their preventable nature^23^. Further studies should assess this variation could be due to changes in nurse-staffing or device-usage. Comparison between international surveillance databases is required to quantify similar associations between climate and gram-negative bacteria, especially considering the rising prevalence of multiple drug resistance and difficult-to-treat infections.

## Supplementary Information

Supplementary Information.

## Data Availability

The data that support the findings of this study are available from the department of Healthcare-Associated Infections and Antimicrobial Resistance within Sciensano. However, restrictions apply to the availability of the data, which were used under license for this current study, and so are not publicly available. Data are however available from the authors upon reasonable request and with permission of the responsible department.
