# Supplementary material for: Gram-negative central line-associated bloodstream infection incidence peak during the summer: a national seasonality cohort study
Source: Sci Rep. 2022 Mar 25;12:5202. doi: 10.1038/s41598-022-08973-9 (PMC8956625; doi:10.1038/s41598-022-08973-9)
Supplement: Supplementary file 1 — Supplementary Information. [file 41598_2022_8973_MOESM1_ESM.docx]

Gram-negative central line-associated bloodstream infection incidence peak during the summer: a national seasonality cohort study

Koen Blot, Naïma Hammami, Stijn Blot, Dirk Vogelaers, Marie-Laurence Lambert

Appendix table of contents

Appendix 1. Surveillance programme bloodstream infection (BSI) case definition

Appendix 2. Surveillance programme central line-associated bloodstream infection (CLABSI) definition criteria

**Appendix 3. Seasonal variation of intensive care unit central line-associated bloodstream infection incidence, per microorganism**

**Appendix 4. Sensitivity analysis of incidence rate ratio variation among central line-associated bloodstream infections with a definite diagnosis, hospital-wide**

Appendix 1. Surveillance programme bloodstream infection (BSI) case definition

| **BSI Criteria** |
| --- |
| Recognized pathogen isolated from ≥1 blood culture |
| Common skin commensal* cultured from ≥2 blood cultures drawn on separate occasions (within 3 days of each other) *and* at least one of the following clinical symptoms within 24 hours of the positive culture:  1. ≥12 months: fever (>38°C), chills, or hypotension  2. <12 months: fever (>38°C), hypothermia (<36.5°C), apnea (5 sec), bradycardia (<80/min) |
| For neonates (≤28 days): ≥1 positive blood culture for coagulase-negative staphylococcus (≥2 days after birth) *and*  1. ≥2 of the following: fever (>38°C), unstable temperature, hypothermia (<36.5°C), tachycardia (>200/min), bradycardia, capillary refill >2 sec, apnea, unexplained metabolic acidosis (BE ≤ 10mEq/L), new-onset hyperglycemia (>140mg/dL), or other signs (skin colour, increased respiratory support, apathy, hemodynamic instability) *and*  2. ≥1 of the following: CRP >2.0mg/dL, neutrophil ratio I/T > 0.2, leukopenia <5/nL, or thrombopenia <100/nL |

* Organisms that constitute normal skin flora include diphteroids, Bacillus sp., Propionibacterium sp., coagulase-negative staphylococci [CoNS], or micrococci.

Appendix 2. Surveillance programme central line-associated bloodstream infection (CLABSI) definition criteria

| **CLABSI Criteria in 2000–2012** |
| --- |
| **Confirmed**: Bloodstream infection with a concomitant positive culture of the catheter tip, using one of the following semi-quantitative methods:  1. ≥15 colony forming units (CFU) in a semi-quantitative culture (24h incubation) on a catheter segment (5–7cm) rolled over a blood agar (roll-plate Maki method)  2. >10^3^ colonies (CFU) per intradermal catheter segment (1cm), washed with a liquid blood agar by ‘flushing’ or ‘vortex’ after semi-quantitative culturing of the flushing medium  3. ‘Paired samples’: the same microorganisms (species and antibiogram) are cultured from a peripheral vein sample and catheter with, in a quantitative culture, the number of CFUs in catheter / number of CFUs peripheral blood >5  **Probable**: However, if one of the three previous criteria are not satisfied but the bloodstream infection is still considered to be related to the central venous catheter, it can be noted as a probable central line-associated bloodstream infection. |
| **CLABSI Criteria in 2013–2014** |
| A central line-associated bloodstream infection is a BSI where a central line was in place during the 2 calendar days before the onset of infection *and* no other cause of infection can be identified |

**Appendix 3.1. Mixed-effects regression analysis of peak-to-low incidence rate ratio seasonality, within the intensive care unit**

| CLABSI |  | IRR | 95% CI | p-value | Peak month |
| --- | --- | --- | --- | --- | --- |
| Total |  | 1.42 | 1.29–1.43 | <0.001 | October |
| Gram-positive |  | 1.24 | 1.10–1.39 | <0.001 | July |
| Gram-negative |  | 3.37 | 2.75–4.11 | <0.001 | October |
| Mixed-effects negative binomial regression model that calculated the intensive care unit central line-associated bloodstream infection (CLABSI) incidence rate ratio (IRR) using hospital units as random effects and university-affiliation status as fixed effects. Incidence rate ratios are expressed as a peak-to-low ratio between the lowest incidence rate in February to the respective peak month per pathogen. *Candida* species seasonality could not be analysed due to insufficient data points. Missing ICU patient day data was interpolated based on the median monthly number of patient days, however this did not influence the results of this analysis. | | | | | |

**Appendix 3.2. Seasonal variation of intensive care unit central line-associated bloodstream infection incidence, per microorganism**

**
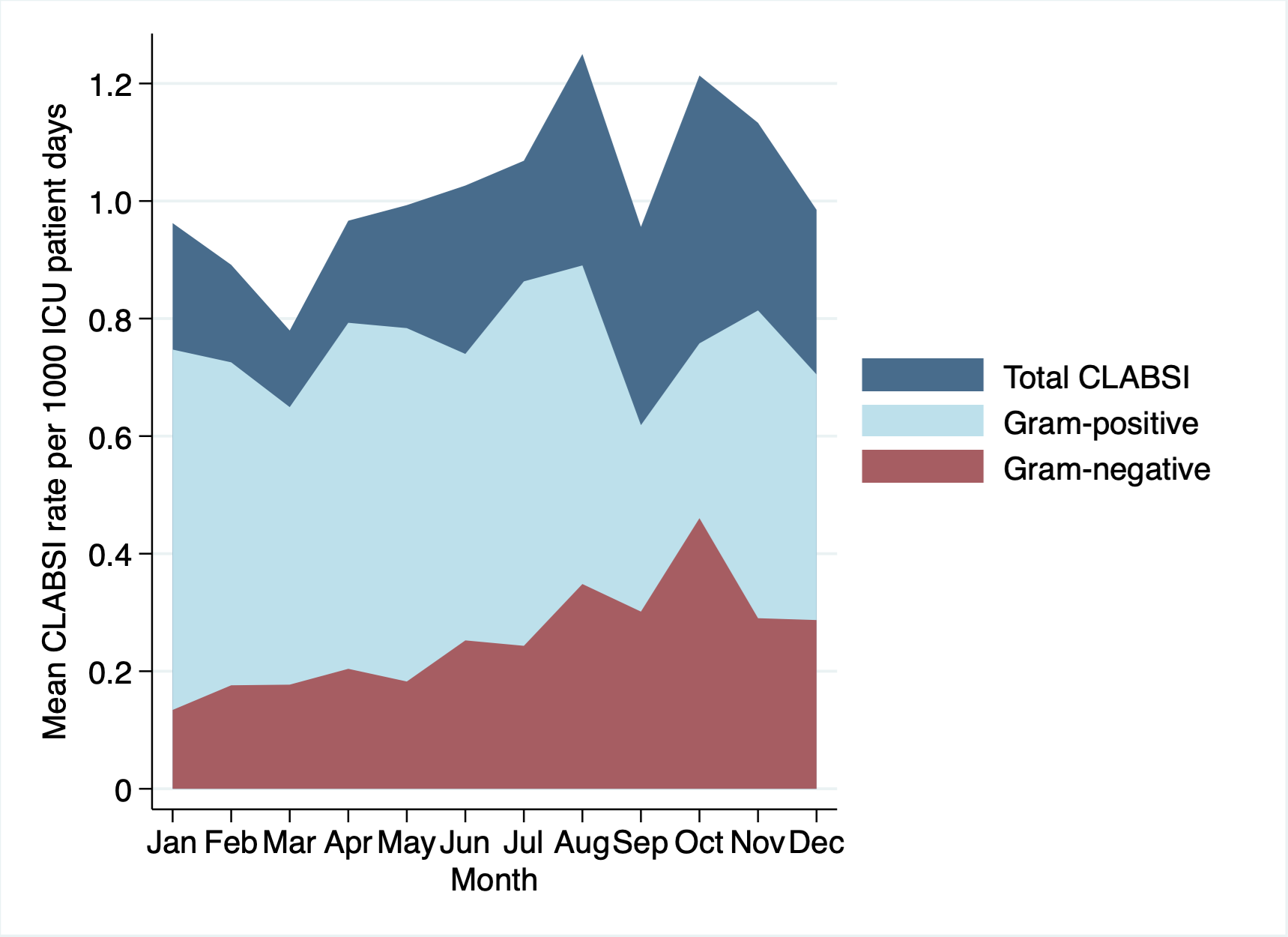
**

*Composite monthly incidence rates of central line-associated bloodstream infection (CLABSI) based on the mixed-effects regression analysis results in the intensive care unit (ICU) (appendix 4). These results demonstrate that the increase of gram-negative CLABSI hospital-wide is replicated in the ICU, yet with a peak following the summer month of August. The rate of total CLABSI classifies polymicrobial BSI as a single CLABSI. Missing ICU patient day data was interpolated based on the median monthly number of patient days, however this did not influence the results of this analysis.*

**Appendix 4. Sensitivity analysis of incidence rate ratio variation among central line-associated bloodstream infections with a definite diagnosis, hospital-wide**

| CLABSI |  | IRR | 95% CI | p-value | Peak month |
| --- | --- | --- | --- | --- | --- |
| Total |  | 1.47 | 1.29–1.67 | <0.001 | August |
| Gram-positive |  | 1.29 | 1.11–1.48 | 0.001 | July |
| Gram-negative |  | 2.52 | 1.92–3.30 | <0.001 | August |
| *Candida* spp. |  | 1.89 | 1.34–2.66 | <0.001 | September |
| From 2000–2012 CLABSI were classified as definite or probable (appendix 2: table 2). Mixed-effects negative binomial regression model that calculated the incidence rate ratio (IRR) using hospital units as random effects and other covariates such as year and university-affiliation as categorical fixed effects. Incidence rate ratios are expressed as a peak-to-low ratio between the lowest incidence rate in February to the respective peak month per pathogen. CLABSI: central line-associated bloodstream infections. | | | | | |
